# A Controlled Challenge Study on Di(2-ethylhexyl) Phthalate (DEHP) in House Dust and the Immune Response in Human Nasal Mucosa of Allergic Subjects

**DOI:** 10.1289/ehp.11474

**Published:** 2008-07-07

**Authors:** Tom Deutschle, Rudolf Reiter, Werner Butte, Birger Heinzow, Tilman Keck, Herbert Riechelmann

**Affiliations:** 1 Department of Otorhinolaryngology, University of Ulm, Medical School, Ulm, Germany; 2 Institute for Pure and Applied Chemistry, University of Oldenburg, Oldenburg, Germany; 3 State Agency for Nature and Environment of Schleswig-Holstein, Department of Environmental Health and Toxicology, Flintbek, Germany; 4 Department of Otorhinolaryngology, University Hospital, Medical University, Innsbruck, Austria

**Keywords:** allergy, cytokines, DEHP, di(2-ethylhexyl) phthalate, house dust, hypersensitivity, microarray analysis, nasal challenge, nasal exposure system, nasal mucosa

## Abstract

**Background:**

Few studies have yet addressed the effects of di(2-ethylhexyl) phthalate (DEHP) in house dust on human nasal mucosa.

**Objectives:**

We investigated the effects of house dust containing DEHP on nasal mucosa of healthy and house dust mite (HDM)–allergic subjects in a short-term exposure setting.

**Methods:**

We challenged 16 healthy and 16 HDM-allergic subjects for 3 hr with house dust at a concentration of 300 μg/m^3^ containing either low (0.41 mg/g) or high (2.09 mg/g) levels of DEHP. Exposure to filtered air served as control. After exposure, we measured proteins and performed a DNA microarray analysis.

**Results:**

Nasal exposure to house dust with low or high DEHP had no effect on symptom scores. Healthy subjects had almost no response to inhaled dust, but HDM-allergic subjects showed varied responses: DEHP_low_ house dust increased eosinophil cationic protein, granulocyte-colony–stimulating factor (G-CSF), interleukin (IL)-5, and IL-6, whereas DEHP_high_ house dust decreased G-CSF and IL-6. Furthermore, in healthy subjects, DEHP concentration resulted in 10 differentially expressed genes, whereas 16 genes were differentially expressed in HDM-allergic subjects, among them anti-Müllerian hormone, which was significantly up-regulated after exposure to DEHP_high_ house dust compared with exposure to DEHP_low_ house dust, and fibroblast growth factor 9, *IL-6*, and transforming growth factor-β1, which were down-regulated.

**Conclusions:**

Short-term exposure to house dust with high concentrations of DEHP has attenuating effects on human nasal immune response in HDM-allergic subjects, concerning both gene expression and cytokines.

Phthalates are a group of organic esters known as indoor air pollutants. The most common phthalate indoors is di(2-ethylhexyl) phthalate (DEHP), which is widely used as plasticizer (softener), for example, to render polyvinyl chloride (PVC) more flexible. A wide variety of consumer products also contain DEHP, such as flooring and other building materials, household furnishings, clothing, cosmetics and personal care products, lubricants, waxes, cleaning materials, and medical products (Schettler 2006). Other phthalates commonly found in house dust include *n*-butyl benzyl phthalate (BBzP), di-*n*-butyl phthalate (DnBP), diisobutyl phthalate (DIBP), and diethyl phthalate (DEP) (Bornehag et al. 2005). Because phthalates are not covalently bound to the plastic matrix, they vaporize directly into the environment, where they can accumulate and adhere to inhalable airborne and sedimented dust particles. In several studies, the median DEHP concentrations in common households ranged from 0.4 to 0.96 mg/g house dust (Bornehag et al. 2005; Butte et al. 2001; Fromme et al. 2004; Kolarik et al. 2008; Pöhner et al. 1997).

Indoor air is an important pathway of human environmental exposure to various phthalates (Latini 2005). One of the potential health concerns of phthalate exposure is the development of asthma and allergies. Emissions from the degradation of PVC flooring materials evoke conjunctival, upper airway, and pulmonary irritations (Jaakkola et al. 1999; Wieslander et al. 1999). In addition, an association between the presence of DEHP in dust and the prevalence of asthma in exposed children has been suggested (Bornehag et al. 2004). Laboratory studies have shown that many phthalate compounds administered to mice by subcutaneous injection or by inhalation exert an adjuvant effect on the immune response to exposure to a coallergen (Hansen et al. 2007; Larsen et al. 2001b, 2001a). After stimulation with DEHP, murine T cells respond with the enhanced production of interleukin (IL)-4 (Lee et al. 2004), whereas cultured neutrophils of both humans and rodents exhibit an inflammatory response (Gourlay et al. 2003). However, few human exposure studies have addressed the effect of phthalates in house dust on mucosal airway response in humans. Tuomainen et al. (2006) challenged 10 subjects experimentally to degraded PVC products under controlled conditions and found no differences in the cytokine expression profiles in the nasal lavage fluid between challenge and control exposure, concluding that PVC materials do not evoke immediate asthmatic reactions. Further evidence for direct health effects of inhalational exposure to DEHP in humans is rare. In the present study, we challenged healthy and allergic human subjects with house dust containing low (0.4 mg/g) and high (2.0 mg/g) concentrations of DEHP in a short-term exposure setting. After nasal dust exposure, we analyzed differences of the transcriptional and secretory response of nasal mucosa using an oligonucleotide cDNA microarray and a microsphere-based flow cytometric assay.

## Materials and Methods

### Subjects and eligibility

Sixteen healthy and 16 house dust mite (HDM)–allergic human subjects participated in this exposure study. Twenty subjects were male, and 12 were female, with ages ranging from 22 to 32 (mean, 24) years. Subjects without clinically relevant nasal disorders, except for allergic subjects suffering from HDM allergy, were eligible, as confirmed by rhinologic history and nasal endoscopy. We identified HDM-allergic subjects by skin prick test according to the guidelines of the European Academy of Allergy and Clinical Immunology (Dreborg and Frew 1993): a CAP [or RAST (radioal-lergosorbent test)] class of at least 3 (Phadia AB, Uppsala, Sweden) and a positive nasal or conjunctival provocation test (Riechelmann et al. 2003b). Exclusion criteria were smoking, pregnancy or lactation, allergy to allergens other than HDM, a clinically relevant nasal septal deviation or turbinate hypertrophy, rhinitis medicamentosa, chronic rhinosinusitis, acute rhinosinusitis within the preceding 6 weeks, previous sinus surgery, bronchial hyperreactivity, lung emphysema, and any systemic therapy with corticosteroids or anti-inflammatory drugs or nasal corticosteroid therapy within the preceding 6 weeks. Our study was approved by the ethics committee of the University of Ulm, and each participant gave written informed consent.

### Study design

**Figure 1:**
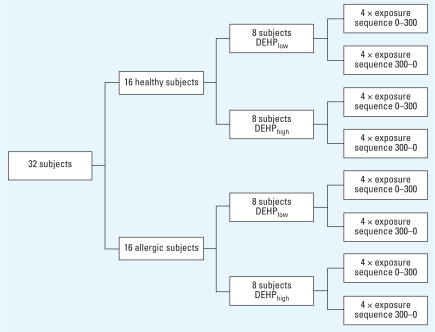
Study flow chart. Healthy and HDM-allergic subjects were randomly arranged for challenge with either DEHP_low_ or DEHP_high_ house dust and the sequence of dust (300 μg/m3) and control (filtered air, 0 μg/m3) exposure.

We randomly assigned subjects to four groups exposed to house dust with either low or high concentrations of DEHP (Figure 1): We challenged eight healthy and eight allergic subjects with DEHP_low_ (0.41 mg/g) house dust, and eight healthy and eight allergic subjects with DEHP_high_ (2.09 mg/g) house dust. We challenged each subject twice in a nose-only exposure setting: once with the house dust in a concentration of 300 μg/m^3^ for 3 hr, and once with filtered air (control, 0 μg/m^3^) for the same time period. The sequences of exposures varied in a random order and were single blinded. We kept a time interval of at least 14 days between the two exposures. We completed visual analogue scales (VASs) immediately before and after exposure. We collected nasal fluid on one nasal side 3 hr after the first exposure, and took a nasal biopsy on the opposite side. After the second exposure, we switched both sides.

### Aerosol exposure

We generated aerosols with a modified rotation plate aerosol generator (Small Scale Powder Disperser, model 3433; TSI GmbH, Aachen, Germany) (Riechelmann et al. 2004). We dispersed the dust in conditioned air (35% relative humidity, 21°C). During the exposures, we constantly recorded the accuracy of dust exposures employing a laser particle counter (Microair-5230; Hiac Royco, Leonberg, Germany).

### House dust

We collected the house dust in 42 households using commercial vacuum cleaners. We sieved the content of the vacuum cleaner bags to a fraction of less than 32 μm and then pooled and divided the dust in two fractions. We used one fraction directly for exposure (DEHP_low_), and enriched the second fraction with DEHP_high_ in a rotary evaporator (Rotavapor R-200; Buchi, Flawil, Switzerland). Thus, the two house dust fractions were identical except for their content of DEHP. We measured the concentrations of DEHP and other phthalates by gas chromatography/mass spectrometry (HP G1800A, GCD series II MS; Agilent Technologies, Santa Clara, California, USA) (Butte et al. 2001).

We determined concentrations of several indoor allergens, including Der p1, Der f1, and Fel d1, with the Dustscreen immunodot assay (CMG Heska, Fribourg, Switzerland). We detected the presence of grass, alder, birch, and yew pollen by light microscopy. Measurements of polycyclic aromatic hydrocarbons, polychlorinated biphenyls, biocides, phenols, heavy metals, tensides, and other contaminants are described elsewhere (Riechelmann et al. 2007).

### Symptom scores

Before and immediately after each exposure, the subjects estimated their sensation of nasal obstruction, hyper-secretion, dryness, itching, and sneezing, mucous membrane burning and unpleasant smell on a 10-cm-long horizontal VAS. We measured the distance from the left border of the scale to the marks made by the subjects in millimeters and calculated the difference after versus before exposure. Positive values indicate a degradation of the appropriate symptom after challenge compared with the control.

### Nasal secretions and biopsy

At 3 hr post-exposure, an open-cell flexible polyurethane foam sampler of 28 × 18 × 6 mm was placed into one nasal cavity posterior to the muco-cutaneous junction under direct visualization and left in place for 10 min. After removal, we extracted the secreted fluid from the sampler by centrifugation and stored it at −20°C (Riechelmann et al. 2003a). On the opposite side, we took a nasal inferior turbinate biopsy under local anesthesia with a Fokkens forceps (Explorent, Tuttlingen, Germany).

### Cytokines and eosinophil cationic protein

We diluted nasal secretions 1:10 and analyzed cytokine concentrations on a Bio-Plex Suspension Array System (Bio-Rad Laboratories, Munich, Germany) for IL-2, IL-4, IL-5, IL-6, IL-8, interferon-γ (IFNγ ), and granulocyte-colony–stimulating factor (G-CSF) employing a multiplex cytokine assay for seven cytokines (Bio-Rad Laboratories) with the Bio-Plex Manager Software 3.0 (Bio-Rad Laboratories). We measured eosinophil cationic protein (ECP) using a fluorescence enzyme immunoassay with the UniCAP 100 Diagnostic System (Phadia, Freiburg, Germany). We performed all assays according to the manufacturers’ recommendations.

### DNA microarray analysis

We performed intraindividual differential (control vs. house dust exposure) gene expression analysis from four subjects of each exposure group. We randomly selected subjects for microarray analysis. The cDNA microarray consisted of 1,232 human genes, 10 different extrahuman spiking controls from *Arabidopsis* and *Sinorhizobium* genes, and randomized negative controls (i.e., oligonucleotides that do not bind human mRNA) in 300 and in 12 spot quadruples as described previously (Riechelmann et al. 2007). Briefly, we spotted oligomers (Operon Biotechnologies, Cologne, Germany) on Ultra Gaps 2 coated slides (Corning, Schiphol-Rijk, The Netherlands). We isolated total RNA from biopsy specimens using the RNeasy Mini Kit (Qiagen, Cologne, Germany). For spiking controls, we synthesized synthetic mRNA of 10 different *Arabidopsis* and *Sinorhizobium* genes on an ABI 394 synthesizer (Purimex, Staufenberg, Germany). We added 0.2–10 pg of this spiking mRNA to the total RNA of control and exposure specimens, resulting in ratios of 1:2 to 1:10. We reverse transcribed mRNA, labeled it using Superscript-2 (Invitrogen, Karlsruhe, Germany) in combination with the 3 DNA Array 350 Kit (Genisphere, Hatfield, PA, USA), and hybridized it with the spotted oligonucleotides at 57°C overnight. We

scanned microarrays with a dual-laser micro-array scanner (GenePix 4000 B; Axon Instruments, Foster City, CA, USA) recorded with GenePix Pro 4.1 software (Axon Instruments) and carried out further analysis using the platform-independent java application ArrayNorm, version 1.7.2 (Pieler et al. 2004). After background subtraction and Lowess subgrid normalization, we performed replicate handling as we averaged quadrupled spots within a slide. Accordingly, we calculated the log_2_-transformed cyanine 5:cyanine 3 ratio (log_2_-expression ratio) for all genes and controls. We identified differentially expressed genes between DEHP_low_ and DEHP_high_ exposure using Student’s *t*-test with Bonferroni step-down correction to adjust the critical alpha limit of 0.05. We considered only genes with valid expression values in each biologic replicate for analysis. We annotated genes according the glossary of the Human Genome Organisation (HUGO) (Wain et al. 2004). The full list of array data is available on the server of the Ear, Nose, and Throat Department Ulm (2008).

### Statistical analysis

For VASs, we calculated changes before and after exposure with the Wilcoxon signed rank test. For protein concentrations, we calculated and tabulated the median and interquartile range (IQR). We calculated dependent samples using the Wilcoxon signed rank test, and independent samples with the Mann–Whitney *U*-test. We considered a *p*-value < 0.05 to be significant. We performed calculations using Systat 10.2 (Systat Software, Point Richmond, CA, USA).

## Results

### House dust

The concentration of DEHP in the DEHP_low_ house dust fraction was 0.41 mg/g. Enrichment of house dust with DEHP yielded to a 5-fold higher concentration of 2.09 mg/g in the DEHP_high_ fraction. Table 1 lists the amounts of phthalates, major allergens, fungal spores, and endotoxin activity. Pollen from weeds, alder, birch, and yew were identified but not quantified.

### Symptom scores

Nasal exposure to DEHP_low_ or DEHP_high_ house dust did not cause significant changes in nasal symptom scores concerning dryness, itching and sneezing, mucous membrane burning, and unpleasant smell compared with filtered air exposure (Table 2). HDM-allergic subjects complained about nasal obstruction and hypersecretion after exposure to DEHP_low_ house dust, but this effect was not significant (*p* > 0.05).

### Cytokines and ECP

Sampling of epithelial lining fluid with a polyurethane foam sampler from all subjects yielded to a median volume of 275 μL (IQR, 180–450 μL). Table 3 outlines the results of protein determination.

**Figure 2:**
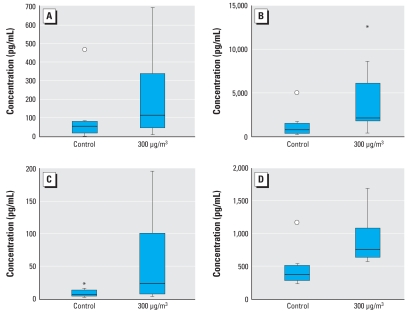
Concentrations of ECP (*A*), G-CSF (*B*), IL-5 (*C*), and IL-6 (*D*) in nasal secretions after nasal exposure of eight HDM-allergic subjects to DEHP_low_ house dust compared with their control exposure to filtered air. *p*-Values calculated with the Wilcoxon signed rank test: ECP, *p* = 0.01; G-CSF, *p* = 0.02; IL-5, *p* = 0.03; IL-6, *p* = 0.02. Horizontal line, median; box contains the central 50% of values; whiskers, observed values; circle, far outside values; asterisk, inner values.

**Figure 3:**
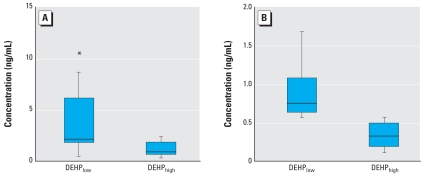
Concentrations of G-CSF (*A*) and IL-6 (*B*) in nasal secretions after nasal challenge of HDM-allergic subjects with DEHP_low_ or DEHP_high_ house dust. *p*-Values calculated with the Mann–Whitney *U*-test: G-CSF, *p* = 0.04; IL-6, *p* = 0.001. Horizontal line, median; box contains the central 50% of values; whiskers, observed values; asterisk, inner values.

Independent from exposure to DEHP_low_ or DEHP_high_ house dust, we found no significant changes in the concentrations of cytokines and ECP in nasal secretions in healthy subjects. Nasal challenge of eight HDM-allergic subjects with DEHP_low_ house dust resulted in significantly elevated median concentrations of ECP (*p* = 0.01), G-CSF (*p* = 0.02), IL-5 (*p* = 0.03), and IL-6 (*p* = 0.02) in nasal secretions compared with the appropriate control exposure with filtered air (Figure 2). The eight HDM-allergic subjects that we challenged with DEHP_high_ house dust showed significantly lower concentrations of G-CSF and IL-6 in nasal secretions than did the HDM-eight allergic subjects we challenged with DEHP_low_ house dust (G-CSF, *p* = 0.04; IL-6, *p* = 0.001) (Figure 3). We found no significant changes for the other cytokines or for ECP.

### DNA microarray gene expression analysis

After control and dust exposures, we obtained nasal mucosa biopsies using the Fokkens forceps from all participants. The median amount of extracted total RNA from biopsy material was 10.1 μg (IQR, 7.8–13.2 μg). We performed gene expression analysis from four subjects of each exposure group. Because of insufficient hybridization quality, we excluded two microarrays from HDM-allergic subjects from further evaluation.

In healthy subjects, we found 10 genes (0.8% of 1,232 genes) to be differentially expressed between the two exposure groups (Table 4). Chemokine (C-C motif) ligand 19 [*CCL19*, UniGene ID NM_006274 (UniGene, http://www.ncbi.nlm.nih.gov/sites/entrez?db=unigene)], a chemokine also known as macrophage inflammatory protein-3β (*MIP-3* β), showed higher expression in healthy subjects after nasal challenge with DEHP_high_ house dust compared with exposure to DEHP_low_ house dust (mean log_2_-expression ratio difference = 0.648; *p* = 0.017).

**Figure 4:**
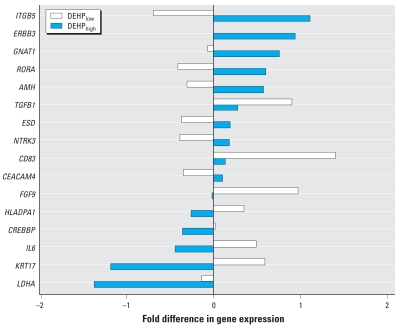
Differentially expressed genes in nasal mucosa of six HDM-allergic subjects exposed to DEHP_low_ (white bars) or DEHP_high_ (black bars) house dust. Values are mean fold difference in log_2_-expression ratios; *p* < 0.05 for all genes. Gene names are abbreviations of the Human Genome Organization (HUGO): *AMH*, anti-Müllerian hormone (NM_000479); *CD83*, CD83 molecule (NM_004233); *CEACAM4*, carcinoem-bryonic antigen-related cell adhesion molecule 4 (NM_001817); *CREBBP*, CREB binding protein (Rubinstein-Taybi syndrome) (NM_004380); *ERBB3*, v-erb-b2 erythroblastic leukemia viral oncogene homolog 3 (NM_001982); *ESD*, esterase D/formylglutathione hydrolase (NM_001984); *FGF9*, fibroblast growth factor 9 (glia-activating factor) (NM_002010); *GNAT1*, guanine nucleotide binding protein (G protein), alpha transducing activity polypeptide 1 (NM_000172); *HLADPA1*, major histocompatibility complex, class II, DP alpha 1 (NM_033554); *IL6*, IL-6 (NM_000600); *ITGB5*, integrin, beta 5 (NM_002213); *KRT17*, keratin 17 (NM_000422); *LDHA*, lactate dehydrogenase A (NM_005566). *NTRK3*, neurotrophic tyrosine kinase, receptor, type 3 (NM_002530); *RORA*, RAR-related orphan receptor A (NM_002943); *TGFB1*, transforming growth factor, beta 1 (NM_000660).

In HDM-allergic subjects, we found 16 genes (1.3% of 1,232 genes) to be differentially expressed between the two exposure groups (Figure 4). We identified eight genes, among them anti-Müllerian hormone (*AMH*, UniGene ID NM_000479), whose expression was significantly higher after exposure to DEHP_high_ house dust compared with exposure to DEHP_low_ house dust (mean log_2_-expression ratio difference = 0.871; *p* = 0.019). We found eight genes whose expression was significantly lower, among them fibroblast growth factor 9 (*FGF9*, NM_002010; 0.975, *p* = 0.016), lactate dehydrogenase A (*LDHA*, NM_005566; 1.237, *p* = 0.013), and the cytokines IL-6 (*IL6*, NM_000600; 0.916, *p* = 0.009) and transforming growth factor-β1 (*TGFB1*, NM_000660; 0.621, *p* = 0.019).

## Discussion

In this study, we investigated the effects of house dust with low and high concentrations of DEHP on nasal mucosa of healthy and HDM-allergic human subjects in a short-term exposure setting. Corresponding parameters included a transcriptional profile of 1,232 genes and the concentrations of several cytokines and ECP in nasal secretions. We found two overall results: *a*) Healthy subjects were not significantly affected by the challenge with house dust, and *b*) HDM-allergic subjects perceived little of the challenge, and low concentrations of DEHP induced a silent inflammation in human nasal mucosa. However, high concentrations of DEHP attenuated the immune response based on protein and mRNA levels.

We collected and pooled house dust from 42 German homes to provide a reasonably representative sample. Coarse particles with diameters > 10 μm are deposited predominantly in the upper airways (Keck et al. 2000). Hence, we used the selected fraction with coarse particles < 32 μm for exposure. A dust concentration of 300 μg/m^3^ is equal to that in interiors with human activity (Junker et al. 2000), in offices (Molhave et al. 2004), and in public transportation (Praml and Schierl 2000). Because of its ubiquity, DEHP appears in almost every house dust sample. Dust samples collected in office buildings in Austria showed DEHP concentrations ranging up to 3 mg/g (Hutter et al. 2006). The median concentration of DEHP in dust collected in children’s bedrooms in 346 homes in Sweden was 0.7 mg/g (Bornehag et al. 2004). These concentrations resemble the concentrations of 0.41 mg/g and 2.09 mg/g used in our study, which consequently agree with DEHP concentrations naturally occurring in house environments. The content of the major allergens Der p1 and Der f1 in house dust depends of several factors, such as geographic region, seasonal variation, climate conditions, age of the dwelling, and floor covering. In dwellings in the Netherlands, van Strien et al. (1994) found a mean Der p1 concentration of 2.4 μg/g for floor dust from living rooms, but in more than the half of the houses the maximum concentration exceeded 10 μg/g. However, the mean concentration of 0.09 μg/g (range, < 0.01–9.54 μg/g) Der p1 found in European homes within the European Community Respiratory Health Survey II is several times lower (Zock et al. 2006). Differences in allergen contents in dust samples may be caused by different dust collecting methods (Schram-Bijkerk et al. 2006). Our house dust was collected over at least 4 weeks in the whole dwelling with conventional vacuum cleaners, provided by the inhabitants themselves. When we used multilayered vacuum cleaner bags, we also included the fine dust fraction between the unique paper layers. It is possible that the long sampling time and dust extracted from the whole vacuum cleaner bag yielded Der p1 and Der f1 concentrations of 2.0 μg/g each, representing typical concentrations in house dust samples from common households. Endotoxin levels resembled concentrations found in a recent European study (Bouillard et al. 2006).

Study subjects’ nasal symptom severity is an important outcome measure in the assessment of dust-induced effects. Symptom scores based on VAS are being used increasingly for this purpose. Concerning healthy volunteers, no upper or lower respiratory symptoms were described after challenge with concentrated ambient air particles (23.1–311.1 μg/m^3^) (Ghio et al. 2000) or urban dust (150 and 500 μg/m^3^) (Riechelmann et al. 2004). However, upper respiratory symptoms were not associated with plastic wall materials (Jaakkola et al. 2000). As expected, house dust did not affect nasal function in our healthy subjects irrespective of the administered DEHP concentration. Although our HDM-allergic subjects had positive nasal or conjunctival provocation tests, nasal challenge with house dust did not result in distinct allergic symptoms. Apparently, the concentration of house dust and the affiliated allergen dose was too low to elicit nasal symptoms. Particularly in persistent allergic rhinitis, affected subjects can habituate to allergen and remain asymptomatic. Among our HDM-allergic subjects, we found a faint but nonsignificant association with increased nasal obstruction and hyper-secretion for DEHP_low_ house dust but not for DEHP_high_ house dust. Phthalates and their metabolites have the potential to interact with the immune system. Concentration-dependent effects of some phthalates are described in animal and *in vitro* studies. In general, lower concentrations of phthalates have shown stimulatory and higher concentrations suppressive properties (Jepsen et al. 2004; Larsen et al. 2002). The effect of DEHP on nasal symptoms of HDM-allergic subjects may be concentration dependent.

Potential adverse effects of phthalates arise from their developmental and reproductive toxicity, particularly in males (Howdeshell et al. 2007). Phthalates act as endocrine disruptors because they alter the hormone profiles of both sexes by interfering with estrogen- or androgen-signaling mechanisms (Ma et al. 2006; Sharpe and Irvine 2004). In a human breast cancer cell line, DEHP mimicked estrogen in the inhibition of tamixofen-induced apoptosis (Kim et al. 2004), and it acts as an anti-androgen in rats (Andrade et al. 2006). *AMH* is a member of the transforming growth factor-β (*TGFB*) family that is implicated in the regression of Müllerian ducts in male fetuses and in the development and function of gonads. Currently, *AMH* is not suspected to play a major role in endocrine disruption (Sharpe 2006). However, in HDM-allergic subjects, transcription of *AMH* increased significantly after exposure to DEHP_high_ house dust compared with DEHP_low_ house dust. It remains unknown whether DEHP affects transcription of *AMH* in nasal mucosa tissue via an androgen or estrogen receptor mechanism or some other pathway. Some more interesting observations include the down-regulation of *LDHA* and *FGF9* in HDM-allergic subjects after exposure to DEHP_high_ house dust. Both genes are involved in sex determination. In the testes of rats, gene expression of *LDHA* is down-regulated after phthalate exposure *in utero* (Plummer et al. 2007) and *FGF9* plays distinct roles in testis development and male sex determination (Colvin et al. 2001). In fact, the potential adverse effects of DEHP have not yet been elucidated in detail. But DEHP is known to be a modulator of gene expression in human cells (Hokanson et al. 2006). Peroxisome-proliferator–activated receptor-α (PPARα) is a member of the nuclear receptor superfamily. Its activation can result in potential anti-inflammatory effects by repressing nuclear factor-κB (NF-κB) (Mandard et al. 2004). Metabolites of DEHP act as ligands of PPARα(Ward et al. 1998). Thus, DEHP may interact with NF-κB via the PPARα-pathway, thereby down-regulating gene expression.

As mentioned above, the effects of some phthalates on immune responses are dose dependent, with adjuvant and suppressive properties in lower and higher concentrations, respectively. Larsen and colleagues demonstrated the adjuvant effect of DEHP after simultaneously administered ovalbumin, indicating that DEHP is a mixed T-helper cell type 1/2 adjuvant (Larsen and Nielsen 2007), but nonoccupational, realistic levels of DEHP neither revealed an adjuvant effect nor induced allergic lung inflammation in mice (Larsen et al. 2007). Our HDM-allergic subjects revealed no significant nasal symptoms after exposure to DEHP_high_ house dust, but the cytokine expression profile changed. We found increases in ECP, G-CSF, IL-5, and/or IL-6 after exposure to DEHP_low_ house dust. After exposure to DEHP_high_ house dust ECP, G-CSF, IL-5, and/or IL-6 were not increased. Additionally, G-CSF and particularly IL-6 decreased significantly after exposure to DEHP_high_ house dust, pointing toward an attenuated immune response at high concentrations of DEHP in house dust. It is not clear from the data in this study whether the applied range of exposure causes a linear or nonlinear attenuation because this study used a three-point design with no exposure (control), DEHP_low_ house dust, and DEHP_high_ house dust. This could be addressed by future studies by having several levels of exposure within a certain range.

As detailed above, a plausible molecular mechanism for the attenuating effects of DEHP is the repression of transcription factor NF-κB. Like DEHP, volatile organic compounds may attenuate the immune response (Wichmann et al. 2005). We found the same diminished release of cytokines after exposure to house dust with high concentrations of DEHP as was found for thalidomide (α-*N*-phthalimidoglutarimide), a sedative with related chemical structure and teratogenic properties (Rowland et al. 1998). However, thalidomide does not act directly on NF-κB (Rowland et al. 2001). These observations do not exclude a possible association between exposure to DEHP and suppressed gene expression via the NF-κB pathway, because the metabolites of DEHP, and not DEHP itself, interact with NF-κB.

The results of our gene expression analysis indicate an altering effect of DEHP on gene expression in human nasal mucosa. The changes demonstrated for IL-6 on the protein level are consistent with the results of gene expression analysis observed in HDM-allergic subjects: *IL-6* was up-regulated after exposure to DEHP_low_ house dust and down-regulated after exposure to DEHP_high_ (Figure 4). We did not detect transforming growth factor-β1 (TGF-β1) protein, but transcription of *TGFB1* decreased significantly after exposure of HDM-allergic subjects to DEHP_high_ house dust. TGF-β1 is an anti-inflammatory cytokine with comparatively high concentrations in healthy mucosa but decreased concentrations in allergic inflammation (Benson et al. 2002). Hence, it remains unknown whether the decrease in *TGFB1* transcription is related to the fact that DEHP attenuates the immune response or to the fact that TGF-β1 is already decreased in allergy mucosa.

## Conclusions

In conclusion, our study provides the first results of short-term exposure to house dust containing low and high concentrations of DEHP on nasal mucosa of healthy and HDM-allergic human subjects. Healthy subjects were not affected by the dust exposures. Low doses of DEHP in house dust elicited a silent inflammation in nasal mucosa of HDM-allergic subjects on both the protein and mRNA level. High concentrations of DEHP in house dust attenuated this inflammation. Our results suggest a stimulating effect at low doses of DEHP and an attenuating effect at high doses. However, exposing adults for 3 hr in a short-term exposure setting is very different from long-term exposure of children to house dust and phthalates in their homes; these two populations may exhibit true differences in immunologic reactions.

## Figures and Tables

**Table 1:** Phthalates, major allergens, fungal spores, and endotoxin activity in house dust.

Compound or allergen	Mean ± SD
Phthalic acid esters (phthalates) (mg/kg)	
*n*-Butyl benzyl phthalate (BBzP)	34.0 ± 4.05
Diisobutyl phthalate (DiBP)	31.7 ± 3.36
Di-*n*-butyl phthalate (DnBP)	49.2 ± 5.7
Diethyl phthalate (DEP)	44.5 ± 4.17
Dimethyl phthalate (DMP)	0.42 ± 0.04
Di-*n*-octyl phthalate (DnOP)	4.23 ± 0.44
Major allergens (μg/g)	
Der p1	2.0 ± 0.1
Der f1	2.0 ± 0.1
Fel d1	5.4 ± 0.5
Fungal spores (cfu/g)	
*Penicillium* spp.	72,000 ± 6,200
*Aspergillus* spp.	8,000 ± 720
Endotoxin activity (EU/g)	15.8 ± 0.6

cfu, colony-forming units.

**Table 2:** Symptom scores (10-cm-long horizontal VAS) after nasal challenge with house dust: difference [median (IQR)] after compared with before exposure.

	DEHP_low_ house dust		DEHP_high_ house dust	
Symptom	Control	Exposure	Control	Exposure
Healthy subjects (*n* = 16)				
OBS	−0.5 (−0.85 to 0.1)	0 (−0.3 to 0.9)	−0.65 (−1.25 to 2.95)	−0.6 (−0.95 to 0.25)
HS	−0.65 (−1.45 to 0.05)	−0.9 (−1.7 to−0.4)	−0.95 (−2.35 to 0.05)	−0.1 (−1.7 to 0.05)
IS	0 (0 to 0.35)	−0.05 (−0.4 to 0)	−0.3 (−1.85 to 0.05)	0 (−0.05 to 0.35)
DRY	0.05 (−0.1 to 0.2)	0.45 (0.2 to 2.35)	1.3 (0.25 to 4)	1.55 (0.3 to 3.2)
SME	0.1 (0 to 0.25)	0.1 (−0.05 to 1.45)	0.05 (−0.05 to 2.05)	0.1 (−0.05 to 2.05)
BUR	0.05 (0 to 0.15)	0.2 (0 to 1.1)	0 (−0.1 to 0.6)	0 (−0.15 to 0.1)
HDM-allergic subjects (*n* = 16)				
OBS	0.05 (−0.75 to 0.45)	1.4 (0.35 to 2.1)	0.15 (−0.25 to 2.35)	−0.15 (−0.75 to 2.6)
HS	−0.45 (−1.3 to 0.1)	0.9 (−0.25 to 2.3)	−0.05 (−1.25 to 0.7)	−0.15 (−1.05 to−0.05)
IS	0 (−0.4 to 0)	0.05 (−0.1 to 0.5)	0.6 (−0.55 to 2.1)	0.75 (−0.05 to 2.35)
DRY	0.2 (−1.15 to 2.6)	1.15 (−0.1 to 3.45)	−0.15 (−1.05 to 3.15)	0.3 (−0.8 to 4.7)
SME	0.05 (−0.1 to 0.1)	0.1 (0 to 1.9)	0.2 (0 to 1.55)	0.2 (0 to 2.05)
BUR	0.05 (0 to 0.15)	0 (−0.05 to 0.1)	0.1 (−0.05 to 0.8)	0.1 (−0.05 to 0.65)

Abbreviations: BUR, mucous membrane burning; HS, hypersecretion; IS, itching and sneezing; DRY, dryness; OBS, sensation of nasal obstruction; SME, unpleasant smell.

**Table 3:** Concentrations of proteins in the epithelial lining fluid of 16 healthy and 16 HDM-allergic subjects after nasal challenge with house dust [median (IQR), rounded to whole numbers].

	DEHP_low_ house dust		DEHP_high_ house dust	
Protein	Control	Exposure	Control	Exposure
Healthy subjects (*n* = 16)				
G-CSF	1,351 (1,049−1,690)	817 (465–2,765)	1,900 (1,128–3,198)	1,656 (319–2,116)
IFNγ	130 (78–180)	147 (119–188)	129 (84–209)	152 (105–230)
IL-2	34 (13–48)	34 (19–59)	19 (4–41)	33 (14–63)
IL-4	118 (66–136)	126 (91–145)	105 (84–138)	117 (79–133)
IL-5	4 (3–41)	5 (2–7)	9 (3–26)	7 (4–15)
IL-6	744 (455–1,201)	572 (289–1,005)	707 (481–1,048)	506 (426–660)
IL-8	1,838 (1,300–2,507)	1,882 (811–2,612)	2,172 (1,165–2,802)	1,402 (1,208–2,114)
ECP (ng/mL)	57 (32–96)	54 (10–156)	60 (10–233)	95 (21–242)
HDM-allergic subjects (*n*= 16)				
G-CSF	793 (313–5,069)	2,165 (482–12,599)	873 (276–4,548)	925 (364–2,432)
IFNγ	99 (78–245)	154 (42–2,497)	139 (47–304)	121 (74–282)
IL-2	30 (9–43)	48 (3–357)	43 (9–107)	28 (9–105)
IL-4	88 (54–153)	121 (66–593)	82 (26–162)	94 (48–156)
IL-5	7 (3–26)	24 (3–195)	16 (4–122)	12 (2–30)
IL-6	373 (232–1,255)	754 (570–1,685)	510 (157–1,537)	332 (113–575)
IL-8	1,835 (971–5,081)	2,778 (788–5,092)	1,611 (661–2,474)	1,851 (563–4,111)
ECP (ng/mL)	57 (2–465)	115 (13–690)	62 (15–327)	106 (19–303)

Values are pg/mL, except where indicated.

**Table 4:** Genes differentially expressed between DEHP_low_ and DEHP_high_ house dust exposure in nasal mucosa of healthy subjects (individual subject values and group means).

	Log_2_-expression ratio for house dust challenge											
	DEHP_low_					DEHP_high_						
HUGO name	24	31	36	40	Mean	22	25	26	34	Mean	Difference of means	*p*-Value
*CCL19* (*)	−0.412	0.182	0.417	−0.040	0.037	0.705	0.211	0.812	0.714	0.611	0.648	0.017
*CREBL1* (*)	−0.129	−0.073	−0.274	0.619	0.036	0.517	1.029	1.559	0.787	0.973	1.009	0.018
*RCN1* (*)	0.955	−0.530	0.085	0.125	0.159	2.200	1.942	1.348	0.430	1.480	1.639	0.024
*SON* (*)	0.077	0.055	0.116	−0.370	−0.031	1.504	1.106	1.733	0.929	1.318	1.349	0.002
*TNFSF10* (*)	−0.269	−0.201	0.372	−1.115	−0.303	0.621	0.975	0.603	0.266	0.616	0.919	0.012
*XCR1* (*)	−0.255	0.243	−0.318	−0.319	−0.162	1.695	0.785	1.714	1.294	1.372	1.534	0.002
*CDKN2B*	0.339	0.087	0.076	0.176	0.170	−0.051	−0.074	−0.008	0.036	−0.024	0.194	0.016
*FCGBP*	0.810	0.472	0.794	−0.079	0.499	−0.075	−0.612	−0.152	−0.269	−0.277	0.776	0.010
*GPX1*	0.076	0.056	0.113	0.098	0.086	0.065	0.039	−0.004	−0.044	0.014	0.100	0.018
*USP20*	−0.085	0.168	0.481	0.331	0.224	−0.636	−0.150	−0.842	−1.592	−0.805	1.029	0.005

Abbreviations: *CCL19*, chemokine (C-C motif) ligand 19 (UniGene ID NM_006274); *CDKN2B*, cyclin-dependent kinase inhibitor 2B (NM_078487); *CREBL1*, cAMP responsive element binding protein-like 1 (NM_004381); *FCGBP*, Fc fragment of IgG binding protein (NM_003890); *GPX1*, glutathione peroxidase 1 (NM_000581); *RCN1*, reticulocalbin 1, EF-hand calcium binding domain (NM_002901); *SON*, SON DNA binding protein (NM_058183); *TNFSF10*, tumor necrosis factor (ligand) superfamily, member 10 (NM_003810); *USP20*, ubiquitin specific peptidase 20 (NM_006676); *XCR1*, chemokine (C motif) receptor 1 (NM_005283). Genes marked with (*) were up-regulated after challenge with DEHP_high_ house dust compared with the challenge with DEHP_low_ house dust; the other genes were down-regulated. Values are log_2_-expression ratios, means, and differences in means; *p*-values are for Student’s *t*-test with Bonferroni step-down correction; gene names are abbreviations of the Human Genome Organization (HUGO).
